# Radiation sensitivity in genetic tumour syndromes and how to test for them

**DOI:** 10.1515/medgen-2025-2041

**Published:** 2025-11-08

**Authors:** Luitpold V. Distel, Laura S. Hildebrand, Lukas C.F. Kuhlmann, Ramona K.G. Vogel

**Affiliations:** University Hospital Erlangen, Friedrich-Alexander-University Erlangen-Nürnberg Department of Radiation Oncology Universitätsstraße 27 91054 Erlangen Germany; University Hospital Erlangen, Friedrich-Alexander-University Erlangen-Nürnberg Department of Radiation Oncology Universitätsstraße 27 91054 Erlangen Germany; University Hospital Erlangen, Friedrich-Alexander-University Erlangen-Nürnberg Department of Radiation Oncology Universitätsstraße 27 91054 Erlangen Germany; University Hospital Erlangen, Friedrich-Alexander-University Erlangen-Nürnberg Department of Radiation Oncology Universitätsstraße 27 91054 Erlangen Germany

**Keywords:** Radiotherapy, Genetic variants, Radiosensitivity, Dose reduction, Late effects, Stochastic risks, BRCA;

## Abstract

Radiation therapy is now well tolerated and associated with few side effects. However, people with certain germline genetic variants may be more sensitive to radiation, increasing their risk of experiencing adverse effects from treatment. Increased sensitivity to radiation can be tested in various ways; chromosomal aberrations, which are mutations, are particularly useful for this purpose. Many genetic variants only cause a slight increase in radiation sensitivity such as heterozygous pathogenic variants in the breast cancer risk genes *BRCA1* and *BRCA2*. Variants of tumour suppressor genes, such as *TP53*, neurofibromatosis (*NF1, PTCH1*) and retinoblastoma (*RB1*), cause a slightly higher increase in radiation sensitivity. However, these also carry a high risk of secondary cancers for only a slightly increased level of risk for therapy-related side effects. Some patients with variants have significantly higher levels of radiation sensitivity – up to double the normal level – while others are even more sensitive. Nevertheless, significant variations exist within each specific genetic disorder. This means that radiosensitivity testing should be considered for all patients with a genetic disorder suspected to markedly increase their radiosensitivity, before they undergo radiotherapy. It implies that patients at risk of germline variants, such as children, young people and others at risk with a tumour, should be carefully evaluated and testing for genetic variants should be performed.

## Introduction

When treating a tumour with ionising radiation (IR) therapy, sufficiently high doses of IR must be used to ensure that all tumour cells are unable to divide. However, this also carries a risk of undesirable side effects in the surrounding normal tissue. Thanks to modern radiation equipment, improved software, and greater computing power, IR can now be targeted more precisely at tumours, sparing normal tissue and reducing the size of radiation fields. Consequently, late side effects in normal tissue caused by radiation are now rare [2]. This highlights other factors that increase the risk of undesirable treatment consequences, one of which is increased radiation sensitivity. Increased radiation sensitivity means that cells are unable to adequately process DNA damage. This involves repairing the damage, transducing signals, and regulating the cell cycle and cell death. If these functions are impaired, a greater number of errors will accumulate, causing the cells to recognise the problem and experience stress. Once a certain trigger value is exceeded, therapy side effects occur. Radiation sensitivity refers to both the stochastic event of cancer development and the more deterministic event of undesirable treatment consequences, with the latter being our main focus. Cancers develop through ionising radiation (IR) when an initial mutation is caused by IR. This increases the probability of further mutations in the cell and its daughter cells. Consequently, the likelihood of mutations being caused by environmental factors increases. Since many mutations must be present in a cell for a tumour to develop, this process can take several years or even decades. However, these two events are inextricably linked. Late side effects can occur within months of treatment or long afterwards, and they are progressive. Radiation sensitivity can be caused by medication, autoimmune diseases, and germline genetic changes [9, 16]. Germline genetic variants that influence radiation sensitivity primarily affect genes involved in DNA repair, signal transduction, and cell cycle regulation [4]. Due to the large number of genes associated with radiosensitivity and the lack of knowledge about the pathogenicity of individual variants, it is not possible to determine radiosensitivity based on germline genetic screening and curation of variants [15]. Therefore, functional tests with endpoints occurring as late as possible are required to determine radiosensitivity. Cell death and radiation induced mutations are late endpoints because they provide information about the processing of DNA damage, the transfer of information within the cell, the regulation of the cell cycle and the control of cell death [18]. Information about radiosensitivity can be obtained either from epidemiological findings, such as an increased incidence of side effects following radiotherapy, or from targeted examinations using functional tests [8].

We outline the testing procedure and provide examples of radiation-sensitive cohorts and the relevant radiation sensitivity syndromes. Additionally, we describe the dose adaptation in radiation therapy for genetic variants.

## Results

### Background of ionizing radiation-induced DNA damage

**Figure 1:**
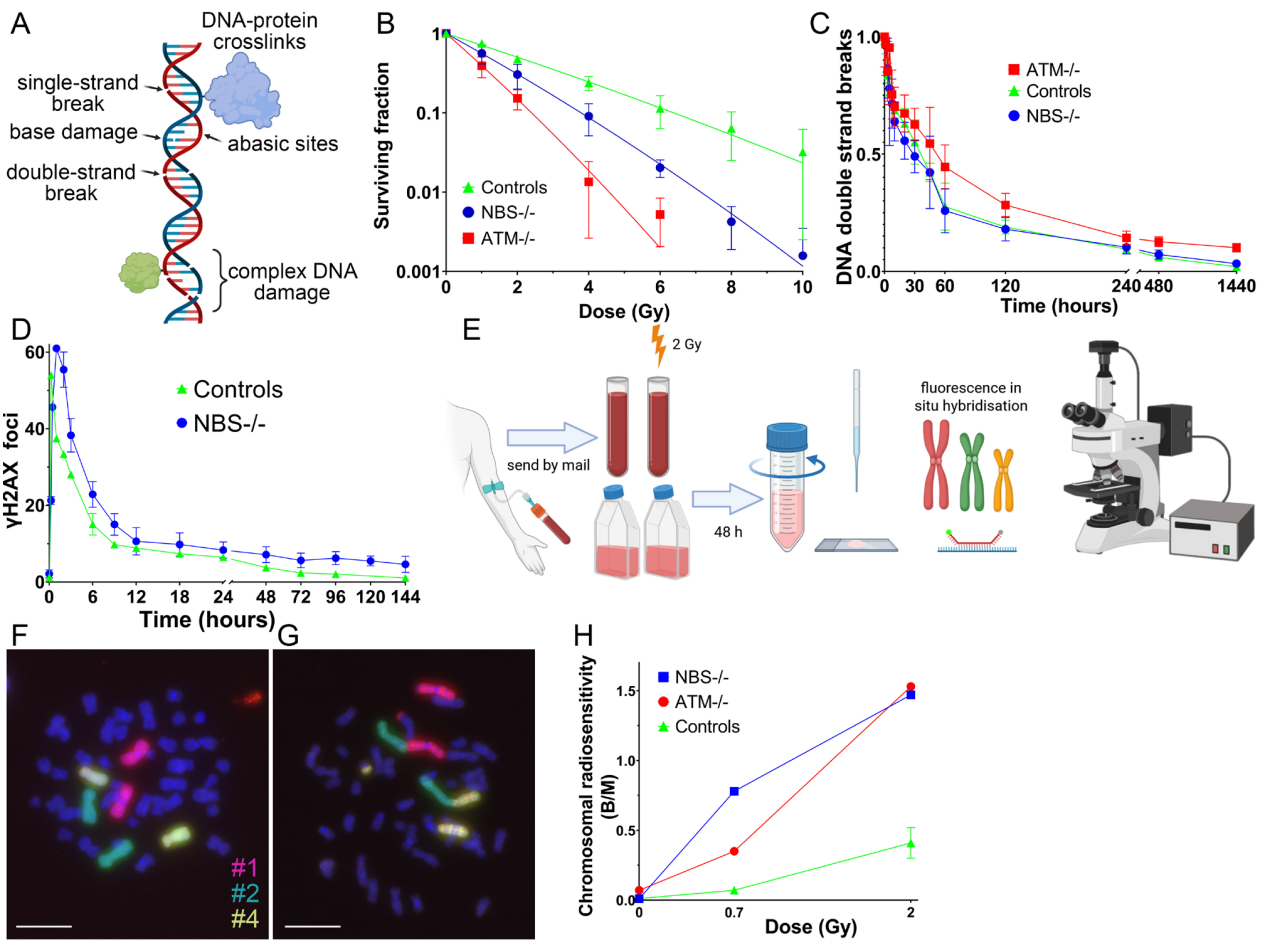
Tests to measure radiation sensitivity. A. DNA damage predominantly caused by ionising radiation. B. Proportion of non-dividing cells after treatment of fibroblasts from control subjects (n = 14), a patient with homozygous Nijmegen breakage syndrome (NBS), and a patient with ataxia telangiectasia mutated (ATM) with increasing dose of IR. C. Measurement of DNA double-strand breaks using constant field gel electrophoresis as a rejoining assay. D. Double-strand break measurement by immunostaining with the γH2AX antibody. E. Radiation sensitivity test procedure: blood sample collection, sent by post, ex vivo irradiation of a blood sample, one blood sample remains unirradiated, blood culture, stimulation of lymphocytes with phytohaemagglutinin, addition of colcemid, preparation of lymphocyte metaphases on slides, staining of chromosomes 1, 2 and 4 with fluorescence in situ hybridisation, recording of metaphases under a fluorescence microscope. F. Metaphase with the three stained chromosomes. G. Metaphase with two translocations. H. Chromosomal aberrations after irradiation of control subjects (n = 14), one patient with homozygous NBS and one patient with ATM.

Ionising radiation causes a wide range of DNA damage, such as base damage and loss, single- and double-strand breaks, and DNA-protein crosslinks. However, this damage can usually be repaired reliably. Complex DNA damage, in which several of the above-mentioned types of damage are present in a small area, is difficult to repair [7]. This damage is specifically caused by IR (Figure 1A). Evidence suggests that limited repair can actually increase a person’s sensitivity to radiation. Moreover, limited signal transduction via the existing damage and impaired cell cycle regulation may also play a role.

### Which method is suitable for measuring radiation sensitivity?

A suitable assay for studying radiation sensitivity is the colony formation test, which shows that cell survival decreases with increasing radiation dose and that different cells differ in their radiation sensitivity [17]. The colony formation assay demonstrates that cells with homozygous mutations in the Nijmegen Breakage Syndrome (NBS) and Ataxia-Telangiectasia Mutated (ATM) genes are extremely sensitive to IR (Figure 1B). The disadvantage is that blood cannot be used for this method and the cultivation of patient-derived skin fibroblasts, which probably could serve as material for a colony formation assay, is too time-consuming to provide timely results for therapy. Alternatively, double-strand break repair can be tested for the determination of radiation sensitivity using blood. But highly radiosensitive *NBS*-/– cells rejoin DNA ends so efficiently that they cannot be distinguished from healthy cells (Figure 1C). When detecting γH2AX foci as a marker for double-strand breaks, a difference between *NBS*-/- and healthy cells can be detected, but the difference underestimates the extreme radiation sensitivity of *NBS*-/– (Figure 1D). We have therefore chosen to use the occurrence of mutations in the form of chromosomal aberrations for detecting radiation sensitivity. It is beneficial that it can be performed with blood, which is easy to obtain and ship. All lymphocytes are naturally present in G0 when irradiated with 2 Gy, which prevents variation in radiation sensitivity due to the cell cycle. Afterwards, the cells are stimulated to divide, can process the DNA damage and migrate once through the entire cell cycle until they are arrested in metaphase by colcemid. In the metaphase, the three largest chromosomes 1, 2 and 4 are stained with fluorescence in situ hybridisation (Figure 1E) and images of the metaphases are recorded (Figure 1F, G). This assay determines radiation sensitivity by counting chromosomal aberrations in approximately 200 metaphases in order to calculate breaks per metaphase (B/M). After 2 Gy irradiation, there are clear differences between healthy individuals and *NBS*-/- and *ATM*-/- individuals (Figure 1H).

## Radiation sensitivity measurement in germline variants using three colour fluorescence in situ hybridization.

**Figure 2:**
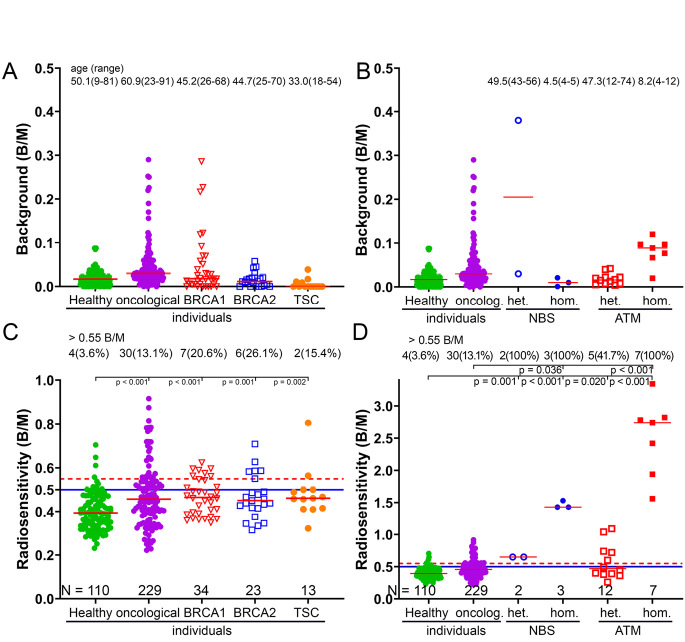
The background of chromosomal aberrations and radiation sensitivity A. Background chromosomal aberrations in a cohort of healthy individuals, patients with oncological diseases, individuals with variants in the *BRCA1*, *BRCA2* or *TSC1* or *TSC2* genes. B. Background in individuals with heterozygous or homozygous variants in the Nijmegen Breakage Syndrome gene or the Ataxia Teleangiectasia gene. C. Chromosomal aberrations after ex vivo irradiation with 2 Gy and as a measure of increased radiation sensitivity in healthy individuals, patients with oncological diseases, individuals with variants in the *BRCA1*, *BRCA2* or *TSC1* or *TSC2* gene. All studied *BRCA1* and *BRCA2* genes were classified as probable pathogenic or pathogenic. D. Radiation sensitivity in individuals with heterozygous or homozygous variants in the Nijmegen Breakage Syndrome gene or the Ataxia Teleangiectasia gene. The average age and range are indicated in the upper section. The number of individuals in each cohort is indicated in the lower section. The solid blue line represents the mean plus twice the standard deviation of the normal distribution, and values above this are considered to be highly sensitive to radiation. Values above the red dotted line are considered to be so highly elevated that the dose should be reduced in the event of radiation therapy. The upper section shows the number of individuals and the percentage with increased radiation sensitivity above 0.55 B/M. Significance values were only calculated for healthy individuals and those with cancer in relation to the syndromes. Values smaller than 0.05 were the only ones entered.

In order to determine radiation sensitivity, an unirradiated blood sample is measured in addition to the irradiated blood sample, and the existing background is subtracted from the irradiated blood sample to obtain only the effect of the IR. In most cases, the background level of chromosomal aberrations is low, averaging from 0.020 B/M ±0.017 in healthy individuals and 0.054 B/M ±0.090 in patients with oncological diseases. Healthy individuals were defined as having no oncological disease, while oncological individuals were defined as having an oncological disease. However, existing germline genetic alterations were not known in either cohort. Patients with heterozygous variants in *BRCA1/2*, tuberous sclerosis (Figure 2A), and biallelic *NBS*-/-, as well as heterozygous *ATM*+/- mutations had similar low background rates. In contrast, only patients with homozygous *ATM*-/- and one heterozygous *NBS* individual had increased background rates. Some patients in the *BRCA1/2*, *NBS* and *ATM* groups had already been diagnosed with cancer prior to testing. All patients with tuberous sclerosis had benign tumours. None of the patients had undergone radiation therapy prior to radiation sensitivity testing. Young people typically have a low level of chromosomal aberrations in their background. However, those who are highly sensitive to radiation have an elevated level even from a young age (Figure 2B). The radiosensitivity of healthy individuals is significantly lower than that of oncology patients (p < 0.001). Patients with heterozygous variants in *BRCA1* or *BRCA2* and patients with tuberous sclerosis are comparable to oncology patients (p > 0.091) and are more sensitive to radiation than healthy individuals (p < 0.049) (Figure 2C) [13, 14]. Radiation-sensitive individuals were defined as those with an elevated value above 0.5 B/M.

Based on our experience, we recommend reducing the dose for patients only above 0.55 B/M, since the doses used in radiation therapy were geared towards individuals who were more sensitive to radiation and more likely to develop late sequelae. This threshold of 0.55 B/M is also an important value for determining the proportion of radiation-sensitive patients. Compared to oncology patients, for whom 13.1 % should have their dose reduced, similar or higher proportions of patients were found among those with *BRCA1* (20.6 %), *BRCA2* (26.1 %) and TSC (15.4 %) (Figure 2C). In both radiation sensitivity syndromes, the heterozygous *NBS*+/- (0.650 B/M) and *ATM*+/– (0.577 B/M) trait carriers already showed elevated values above 0.55 B/M, with 100 % and 41.7 % respectively. In both syndromes, homozygous trait carriers with *NBS*-/- and *ATM*-/– exhibited extreme radiation sensitivity, with values of 1.46 B/M and 2.51 B/M, respectively (Figure 2D).

### Genetic disorders that are relevant in radiation therapy and associated with an increased risk of radiation sensitivity.

In addition to these syndromes shown as examples, there are a large number of genetic variants that are associated with increased radiation sensitivity (Table 1). The classification is sorted in ascending order for the genes for which we already have experience with radiation sensitivity. However, it must always be borne in mind that there are significant variations within each group and that it is therefore not possible to draw conclusions about individuals. One difficult question is which patients should be tested as part of the daily routine. One group that clearly needs to be tested is those for whom ‘dose adjustment is absolutely necessary’ (Table 1). Otherwise, any type of radiation therapy could have life-threatening consequences, resulting in death [1, 5]. The only other option here would be to forego radiation therapy altogether.

**Figure 3:**
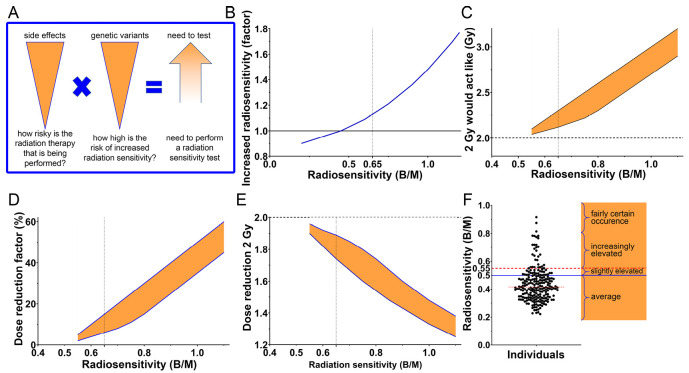
Selection of patients for testing for radiation sensitivity and adjustment of radiation doses for radiotherapy. A. Basic algorithm for determining the need for radiation sensitivity testing. The risk of late side effects from radiation therapy must be considered alongside the estimated level of radiation sensitivity in genetic variants. This combination determines the need for radiation sensitivity testing. B. Correlation between radiation sensitivity determined by chromosomal aberrations and the correlation with radiation sensitivity developed by us. C. The expected effect of a 2 Gy fraction on the correspondingly radiation-sensitive person. D. The resulting recommended dose reduction factor. E. The recommended dose reduction for radiation-sensitive individuals compared to a 2 Gy fraction for an average radiation-sensitive individual. F. Assessment of the significance of the measured radiation sensitivity for the risk of developing late side effects.

For other risk levels with a lower risk of increased radiation sensitivity, the type of radiation therapy used must also be considered. As an example, adjuvant radiation therapy for breast cancer is generally well tolerated and carries a low risk of side effects. In this case, even moderate increased sensitivity to radiation is unlikely to affect the treatment. However, radiotherapy that requires high efficacy, such as that used for head and neck tumours, carries an increased risk of late side effects. In such cases, moderately increased sensitivity to radiation significantly increases the risk of side effects. Additionally, large radiation fields must always be considered, as they also increase the risk of side effects. Ultimately, the risk of radiation therapy causing side effects must be assessed, as well as the probability of increased radiation sensitivity. The urgency of radiation sensitivity testing is determined by combining these two factors (Figure 3A). However, since genetic variants are almost always associated with rare diseases, there is limited empirical data available to provide clear guidance in this area. Testing such patients would be important in order to gain more empirical data. We are always willing to test patients who are at an increased risk, provided they meet the above criteria and request it. Further, patients with genetic variants that only slightly increase radiation sensitivity generally tolerate radiation therapy well, so testing them is usually unnecessary. It is unclear whether pathological variants lead to increased radiation sensitivity. Other indicators, such as the patient or their relatives having previously poorly tolerated radiation therapy, would certainly be reasons to test a patient with a variant that only slightly increases the risk. In addition, it must be borne in mind that individuals affected with disorders associated with a pathogenic variant in a tumour suppressor gene can have a very high stochastic risk, significantly increasing the likelihood of further tumours and secondary malignancies developing as a result of radiotherapy.

**Table 1:** Genetic radiation sensitivity syndromes associated with radiotherapy

Severity of radiation sensitivity	Genotype	Radiation sensitivity in genetic variants listed in ascending order according to severity of radiation sensitivity (own research) [6, 19]
Slightly increased	-	Goldenhar syndrome, §
	hetero	Hailey-Hailey disease, §
	hetero	*CHEK2*
	hetero	*ATM*
	hetero	*BRCA2*
	hetero	*BRCA1,* ***
	hetero	*NBS*
	hetero	*RAD51*
	hetero	*BARD1*, §
	hetero	Tuberous sclerosis (TS); tuberous sclerosis complex (*TSC1* or *TSC2*), ***
Slight dose adjustment required	hetero	Neurofibromatosis type 1 – Von Recklinghausen syndrome (*NF1*), ***, $
	hetero	Neurofibromatosis type 2 – Gorlins syndrome (*PTCH1*), $
	hetero	*RB1* (*retinoblastoma*), ***
	hetero	Marfan syndrome (MFS), §
	hetero	Li Fraumeni syndrome (*TP53*) ***, $
	somatic	McCune-Albright syndrome (MAS), §
	hetero	Brooke-Spiegler syndrome (BSS), §
Dose adjustment is absolutely necessary; otherwise, it is life-threatening	homo	Bloom’s syndrome (*BLM*), $
	hetero	Phelan-McDermid syndrome (deletion syndrome 22q13, PMS) [12], §, $
	hetero	Lynch syndrome (HNPCC), $
	homo	Fanconi anaemia, $
Dose adjustment is absolutely necessary; otherwise, it is not survivable	homo	PNKP mutation, §, $
	homo	LIG4 mutation – Ligase IV syndrome – Lig4 syndrome, $
	homo	Nijmegen breakage syndrome (*NBS*), $
	homo	Ataxia Teleangiectasia Mutated (*ATM*), $
Further genetic variants listed alphabetically (without personal experience)	homo	ATLD (*MRE11*), $
	homo	Cockayne syndrome (A, B, C)
	hetero	Gardner’s syndrome (APC)
	homo	Glutathione synthetase deficiency (GSS)
	hetero	Hutchinson-Gilford progeria syndrome (LMNA)
	homo	Hypogammaglobulinemia Lig I deficiency
	homo	ICF syndrome (DNMT3B)
	homo	NBSLD syndrome (*RAD50*), $
	somatic	Proteus syndrome
	hetero	Rett syndrome (MeCP2 (methyl-CpG-binding protein 2), Xq28)
	homo	Rothmund-Thomson syndrome (*RECQL4* gene / helicase)
	homo	SCAN1 (spinocerebellar ataxia with axonal neuropathy, tyrosyl-DNA phosphodiesterase (Tdp1), removed blocked 3′-termini in DNA strand breaks., $
	homo	SCID (Artemis), $
	homo	SCID-DNA-PK defect (immunodeficiency), $
	homo	Trichothiodystrophy (TTD, excision repair system)

*** Germline genetic variants with high stochastic risk compared to deterministic risk

§ Assessment of radiation sensitivity based on own measurements.

$ We strongly recommend testing for individual radiosensitivity prior to radiotherapy, as based on literature and our own research.

hetero = heterozygous variant; homo = homozygous variant; somatic = somatic mutation

### Adjustment of radiation therapy in patients with increased sensitivity to radiation

To take into account the dynamic development of newly discovered genetic variants associated with radiation sensitivity, we have created a website where we regularly publish the results of our radiation sensitivity studies (https://www.strahlenklinik.uk-erlangen.de/forschung/strahlenempfindlichkeitssyndrome-und-erkrankungen/). Therefore, it is also important to test patients with genetic variants for which it is not known whether, or to what extent, they cause radiation sensitivity, in order to increase our knowledge of radiation sensitivity in rare diseases. The testing process takes two weeks, but can be shortened to six days if radiation therapy needs to start very urgently. The contribution of our research to radiation therapy is the patient-specific quantification of increased radiation sensitivity calculated by the chromosomal radiation sensitivity, expressed as B/M. This correlation was determined empirically (Figure 3B). This means that, for patients with increased radiation sensitivity, each fraction of the dose has a stronger effect on cancerous and normal tissue than it would for patients with average radiation sensitivity (Figure 3C). Therefore, the fraction dose must be reduced by a factor depending on the radiation sensitivity (Figure 3D). This reduced dose is equally effective for normal tissue and cancer in a highly radiosensitive person as it is for someone with average radiosensitivity (Figure 3E). In a small study on increased radiation sensitivity caused by the autoimmune disease lupus erythematosus, we were able to show that reducing the dose reduces undesirable side effects of treatment and that no recurrences occurred during the short follow-up period [16]. To illustrate the relationship between radiation sensitivity and the risk of late side effects, we will attempt to categorise the importance of different levels of radiation sensitivity. We would expect an average risk of late side effects for people with average radiosensitivity, and thus a very low risk. In contrast, for radiosensitive patients the risk for severe side effects is increased if the dose is not adjusted. In the range from 0.5 to 0.55 B/M, we would expect a slightly increased risk, but this is still so low that no dose adjustment is necessary. According to the above model, we would recommend adjusting the dose for planned radiation therapy if the measured radiosensitivity value is above 0.55 B/M (Figure 3F). In our testing, we assume that between 0.55 and 0.75 B/M there is still a stochastic risk of late side effects, which then transitions to a deterministic range at higher B/M values. This means that radiation therapy in this area will certainly lead to undesirable side effects.

However, it should be noted that increased radiation sensitivity always increases the risk of stochastic effects, especially cancer [21]. If a genetic variant that significantly increases the stochastic risk of secondary carcinomas is present, this risk should be taken into account when planning radiotherapy. This is particularly relevant for younger patients, given that it takes at least five to ten years or longer for a secondary carcinoma to develop, and the risk increases throughout life [3]. There appears to be a particularly strong correlation between measured radiation sensitivity and stochastic risk for syndromes that interfere with the repair of radiation-induced DNA damage. Good examples of this are individuals with homozygous variants in the *NBS* and *ATM* genes, both of which are associated with an extremely high stochastic risk and a high risk of adverse treatment outcomes. By contrast, *BRCA1* exhibits only a modest increase in radiation sensitivity and a slightly elevated risk of late side effects, coupled with a high stochastic risk [10]. The tumour suppressor genes *TP53* (Li-Fraumeni syndrome), *RB1* (retinoblastoma), *NF1* (Von Recklinghausen syndrome), *PTCH1* (Gorlin-Goltz syndrome) and *TSC1* or *TSC2* (tuberous sclerosis) are associated with a very high stochastic risk, but mostly only with a limited increased risk of late effects [20]. Given the high level of stochastic risk associated with these syndromes, the need for radiation therapy must be carefully considered.

## Conclusion

In children, young people, and other risk groups with tumours, a careful evaluation should be carried out and genetic testing for germline variants should be performed. Patients with genetic variants that are suspected to significantly increase radiation sensitivity should undergo testing for radiation sensitivity before receiving radiation therapy.

## References

[1] Bakhshi S., Cerosaletti K. M., Concannon P., Bawle E. V., Fontanesi J., Gatti R. A., Bhambhani K. (2003). “Medulloblastoma with adverse reaction to radiation therapy in nijmegen breakage syndrome.”. J Pediatr Hematol Oncol
25(3).

[2] Barazzuol L., Coppes R. P., Luijk P. van (2020). “Prevention and treatment of radiotherapy-induced side effects.”. Mol Oncol
14(7).

[3] Berrington de Gonzalez A., Curtis R. E., Kry S. F., Gilbert E., Lamart S., Berg C. D., Stovall M., Ron E. (2011). “Proportion of second cancers attributable to radiotherapy treatment in adults: a cohort study in the US SEER cancer registries.”. Lancet Oncol
12(4).

[4] Chistiakov D. A., Voronova N. V., Chistiakov P. A. (2008). “Genetic variations in DNA repair genes, radiosensitivity to cancer and susceptibility to acute tissue reactions in radiotherapy-treated cancer patients.”. Acta Oncol
47(5).

[5] Distel L., Neubauer S., Varon R., Holter W., Grabenbauer G. (2003). “Fatal toxicity following radio- and chemotherapy of medulloblastoma in a child with unrecognized Nijmegen breakage syndrome.”. Med Pediatr Oncol
41(1).

[6] El-Nachef L., Al-Choboq J., Restier-Verlet J., Granzotto A., Berthel E., Sonzogni L., Ferlazzo M. L., Bouchet A., Leblond P., Combemale P., Pinson S., Bourguignon M., Foray N. (2021). “Human Radiosensitivity and Radiosusceptibility: What Are the Differences?”. Int J Mol Sci
22(13).

[7] Frey B., Borgmann K., Jost T., Greve B., Oertel M., Micke O., Eckert F. (2023). “DNA as the main target in radiotherapy-a historical overview from first isolation to anti-tumour immune response.”. Strahlenther Onkol
199(12).

[8] Habash M., Bohorquez L. C., Kyriakou E., Kron T., Martin O. A., Blyth B. J. (2017). “Clinical and Functional Assays of Radiosensitivity and Radiation-Induced Second Cancer.”. Cancers (Basel)
9(11).

[9] Hecht M., Zimmer L., Loquai C., Weishaupt C., Gutzmer R., Schuster B., Gleisner S., Schulze B., Goldinger S. M., Berking C., Forschner A., Clemens P., Grabenbauer G., Muller-Brenne T., Bauch J., Eich H. T., Grabbe S., Schadendorf D., Schuler G., Keikavoussi P., Semrau S., Fietkau R., Distel L. V., Heinzerling L. (2015). “Radiosensitization by BRAF inhibitor therapy-mechanism and frequency of toxicity in melanoma patients.”. Ann Oncol
26(6).

[10] Hu C., Hart S. N., Gnanaolivu R., Huang H., Lee K. Y., Na J., Gao C., Lilyquist J., Yadav S., Boddicker N. J., Samara R., Klebba J., Ambrosone C. B., Anton-Culver H., Auer P., Bandera E. V., Bernstein L., Bertrand K. A., Burnside E. S., Carter B. D., Eliassen H., Gapstur S. M., Gaudet M., Haiman C., Hodge J. M., Hunter D. J., Jacobs E. J., John E. M., Kooperberg C., Kurian A. W., Le Marchand L., Lindstroem S., Lindstrom T., Ma H., Neuhausen S., Newcomb P. A., O’Brien K. M., Olson J. E., Ong I. M., Pal T., Palmer J. R., Patel A. V., Reid S., Rosenberg L., Sandler D. P., Scott C., Tamimi R., Taylor J. A., Trentham-Dietz A., Vachon C. M., Weinberg C., Yao S., Ziogas A., Weitzel J. N., Goldgar D. E., Domchek S. M., Nathanson K. L., Kraft P., Polley E. C., Couch F. J. (2021). “A Population-Based Study of Genes Previously Implicated in Breast Cancer.”. N Engl J Med
384(5).

[12] Jesse S., Kuhlmann L., Hildebrand L. S., Magelssen H., Schmaus M., Timmermann B., Andres S., Fietkau R., Distel L. V. (2023). “Increased Radiation Sensitivity in Patients with Phelan-McDermid Syndrome.”. Cells
12(5).

[13] Kuhlmann L., Stritzelberger J., Fietkau R., Distel L. V., Hamer H. M. (2024). “Radiosensitivity in individuals with tuberous sclerosis complex.”. Discov Oncol
15(1).

[14] Mayo T., Schuster B., Ellmann A., Schmidt M., Fietkau R., Distel L. V. (2019). “Individual Radiosensitivity in Lung Cancer Patients Assessed by G0 and Three Color Fluorescence in Situ Hybridization.”. OBM Genetics
3(2).

[15] Pearl L. H., Schierz A. C., Ward S. E., Al-Lazikani B., Pearl F. M. (2015). “Therapeutic opportunities within the DNA damage response.”. Nat Rev Cancer
15(3).

[16] Schenker H., Kuhlmann L., Kaudewitz D., Schuster B., Semrau S., Schmitter C., Voigt R., Merten R., Geinitz H., Fietkau R., Boltz S., Schett G., Distel L. V. (2025). “Increased Sensitivity to Ionizing Radiation in a Relevant Subset of Patients with Cancer and Systemic Lupus Erythematosus.”. Cells
14(8).

[17] Serrano-Mendioroz I., Garate-Soraluze E., Rodriguez-Ruiz M. E. (2023). “A simple method to assess clonogenic survival of irradiated cancer cells.”. Methods Cell Biol
174.

[18] Sia J., Szmyd R., Hau E., Gee H. E. (2020). “Molecular Mechanisms of Radiation-Induced Cancer Cell Death: A Primer.”. Front Cell Dev Biol
8.

[19] Tam A., Mercier B. D., Thomas R. M., Tizpa E., Wong I. G., Shi J., Garg R., Hampel H., Gray S. W., Williams T., Bazan J. G., Li Y. R. (2023). “Moving the Needle Forward in Genomically-Guided Precision Radiation Treatment.”. Cancers (Basel)
15(22).

[20] Wood M. E., Vogel V., Ng A., Foxhall L., Goodwin P., Travis L. B. (2012). “Second malignant neoplasms: assessment and strategies for risk reduction.”. J Clin Oncol
30(30).

[21] Zamboglou C., Aebersold D. M., Albrecht C., Boehmer D., Ganswindt U., Schmidt-Hegemann N. S., Hoecht S., Holscher T., Koerber S. A., Mueller A. C., Niehoff P., Peeken J. C., Pinkawa M., Polat B., Spohn S. K. B., Wolf F., Zips D., Wiegel T. (2025). “The risk of second malignancies following prostate cancer radiotherapy in the era of conformal radiotherapy: a statement of the Prostate CancerWorking Group of the German Society of Radiation Oncology (DEGRO).”. Strahlenther Onkol
201(1).

